# Genetic parameters for milk fatty acids in Danish Holstein cattle based on SNP markers using a Bayesian approach

**DOI:** 10.1186/1471-2156-14-79

**Published:** 2013-09-11

**Authors:** Kristian Krag, Nina A Poulsen, Mette K Larsen, Lotte B Larsen, Luc L Janss, Bart Buitenhuis

**Affiliations:** 1 Department of Molecular Biology and Genetics, Faculty of Science and Technology, Aarhus University, PO Box 50, DK-8830 Tjele, Denmark; 2 Department of Food Science, Faculty of Science and Technology, Aarhus University, PO Box 50, DK-8830 Tjele, Denmark

**Keywords:** Genomic heritability, Genomic correlation, Bayesian mixed model, Milk fatty acids

## Abstract

**Background:**

For several years, in human nutrition there has been a focus on the proportion of unsaturated fatty acids (UFA) and saturated fatty acids (SFA) found in bovine milk. The positive health-related properties of UFA versus SFA have increased the demand for food products with a higher proportion of UFA. To be able to change the UFA and SFA content of the milk by breeding it is important to know whether there is a genetic component underlying the individual FA in the milk. We have estimated the heritability for individual FA in the milk of Danish Holstein. For this purpose we used information of SNP markers instead of the traditional pedigree relationships.

**Results:**

Estimates of heritability were moderate within the range of 0.10 for C18:1 *trans-*11 to 0.34 for C8:0 and C10:0, whereas the estimates for saturated fatty acids and unsaturated fatty acids were 0.14 and 0.18, respectively. Posterior standard deviations were in the range from 0.07 to 0.17. The correlation estimates showed a general pattern of two groups, one group mainly consisting of saturated fatty acids and one group mainly consisting of unsaturated fatty acids. The phenotypic correlation ranged from −0.95 (saturated fatty acids and unsaturated fatty acids) to 0.99 (unsaturated fatty acids and monounsaturated fatty acids) and the genomic correlation for fatty acids ranged from −0.29 to 0.91.

**Conclusions:**

The heritability estimates obtained in this study are in general accordance with heritability estimates from studies using pedigree data and/or a genomic relationship matrix in the context of a REML approach. SFA and UFA expressed a strong negative phenotypic correlation and a weaker genetic correlation. This is in accordance with the theory that SFA is synthesized *de novo*, while UFA can be regulated independently from the regulation of SFA by the feeding regime.

## Background

For several years, in human nutrition there has been a focus on the proportion of unsaturated fatty acids (UFA) and saturated fatty acids (SFA) found in bovine milk. The positive health-related properties of UFA versus SFA have increased the demand for food products with a higher proportion of UFA. Currently, milk fat contains typically 70% SFA [1] which is high compared to the recommended maximal SFA intake of 7% of total energy intake in a human diet for a healthy lifestyle [2]. Decreasing SFA and increasing UFA in milk fat has shown health benefits and it has been suggested that a target for changing milk fat composition should focus on the reduction of C16:0 [1]. The fatty acid (FA) composition in milk is a result of the genetic composition of the individual cows, lactation stage, feeding, microbial composition in the rumen, and season [3-5].

Most of the short and medium chain FAs (*i.e*., C4:0 to C14:0) are mainly synthesized in the mammary gland, whereas C16:0 FAs are both synthesized in the mammary gland and obtained from food. In contrast, long chain FAs mainly originate from feed, but may be modified in the rumen [6]. This is reflected in previous studies, where the genetic influence is highest for the SFA, whereas UFA is more affected by feed [7,8].

To change the FA composition through selective breeding information is needed whether there is genetic variance for the trait of interest. It has been shown that there are differences between breeds in the proportion of individual FAs within the milk [9]. However, for breeding purposes, the genetic variation within the breed of interest would be informative. Genetic variation for FAs within American, Italian, and Dutch Holstein populations has previously been elucidated [3,4,10]. These studies showed that there was a pattern among FAs, where SFA in general have a higher heritability than UFA. In addition, the genetic correlation between FAs was higher within SFA than within UFA. Correlation between most of the SFA and UFA were strongly positive, whereas the correlation between SFA and UFA were strongly negative.

Most of the studies that have previously estimated genetic parameters, such as the heritability, were based on half-sib groups and pedigree information e.g. [3,4,10]. Recently, Krag et al. [11] showed in a simulation study, that the heritability could be estimated from a sample size of 400 animals based on SNP markers using a Bayesian approach. The authors found that the SNP markers captures the population structure well and that SNP markers could be used as an alternative to traditional pedigree based methods. The aim of this study was to estimate variance components for individual FA and groups of FA as well as covariances among FA and groups of FA in Danish Holstein cattle. Variance components were obtained by simultaneously estimating the variance explained by a panel of SNP markers distributed along the genome by means of a Bayesian mixed model.

## Results

Mean, standard deviation, coefficient of the variation (CV), median, heritability, and posterior standard deviations for the heritability estimates for each trait are presented in Table 1. Mean fat content in the milk was 3.99%. In this study, 17 specific FAs were identified, which comprise 90.16% of the fat content. The remaining 9.84% were fatty acids that were excluded because concentrations were low or separations were poor i.e. the peaks were overlapping with the major FA. The least abundant fatty acid that was measured was C13:0, which represented 0.1% of the fat content. The most abundant FA was C16:0, which comprised 28.95% of the fat. Together with C14:0, C18:0, and C18 *cis*-9, C16:0 accounted for 70.56% of the total fat content, meaning that the other 13 fatty acids combined accounted for 19.6% of the fat content. For single UFA, the CV ranged from 18.04% to 33.27%. For single SFA, the range of CV was 11.23% to 30.27%. In general, high values for CV were found for single FAs that were present in low concentrations. For groups of FAs and the FA indices, the CV ranged from 6 to 15.93 and from 12.24 to 25.02, respectively.

**Table 1:** **Mean, standard deviation (SD) of the mean, coefficient of variation (CV) of the mean, median, genomic heritability estimate (h**_**g**_^**   2**^**), posterior standard deviation (Psd), and Phenotypic variation (Pvar) for the content of fat (g/100 g milk) and fatty acids (g/100 g fat)**

**Trait**	**Mean**	**SD**	**CV**	**Median**	**h**_**g**_^**2**^	**Psd**	**Pvar**
Fat	3.99	0.78	19.64	3.96	0.24	0.13	0.67
**Fatty acids**							
C6:0	2.69	0.35	12.97	2.72	0.24	0.13	0.12
C8:0	1.47	0.23	15.37	1.48	0.34	0.15	0.05
C10:0	3.16	0.56	17.87	3.17	0.34	0.16	0.32
C12:0	3.57	0.65	18.14	3.55	0.27	0.15	0.43
C13:0	0.10	0.03	30.27	0.10	0.19	0.13	0.001
C14:0	11.29	1.30	11.49	11.36	0.25	0.17	1.66
C15:0	1.10	0.20	17.89	1.09	0.13	0.10	0.04
C16:0	28.95	3.25	11.23	28.84	0.14	0.11	0.34
C17:0	0.53	0.15	28.45	0.53	0.07	0.06	0.02
C18:0	10.54	2.20	20.89	10.26	0.19	0.13	4.15
C14:1 *cis-*9	0.97	0.28	29.03	0.94	0.26	0.14	0.07
C16:1 *cis-*9	1.50	0.38	25.14	1.46	0.16	0.11	0.15
C18:1 *trans*-11	1.70	0.56	33.27	1.62	0.09	0.08	0.27
C18:1 *cis-*9	19.78	3.62	18.29	19.83	0.11	0.10	8.46
C18:2 n-6	1.69	0.30	18.04	1.65	0.17	0.11	0.08
C18:3 n-3	0.49	0.10	20.46	0.49	0.30	0.15	0.01
CLA *cis*-9. *trans*-11^1^	0.63	0.16	26.22	0.60	0.19	0.13	0.02
**Groups of fatty acids**							
SFA^2^	63.40	3.81	6.00	63.51	0.09	0.08	11.97
UFA^3^	26.76	3.87	14.46	26.54	0.33	0.19	11.33
MUFA^4^	23.95	3.72	15.52	23.84	0.34	0.20	9.85
PUFA^5^	2.81	0.45	15.93	2.76	0.28	0.15	0.19
C6:0 to C14:0^6^	22.18	2.80	12.63	22.26	0.28	0.17	7.94
**Fatty acids indices**							
C14 index^7^	0.08	0.02	25.02	0.08	0.31	0.16	0.002
C16 index^8^	0.05	0.01	21.53	0.05	0.19	0.12	3.80
C18 index^9^	0.65	0.08	12.24	0.66	0.35	0.19	1.12
CLA index^1.10^	0.28	0.06	22.21	0.27	0.16	0.12	18.55

^1^CLA = conjugated linoleic acid.

^2^Saturated fatty acids: C6:0, C8:0, C10:0, C12:0, C13:0, C14:0, C15:0, C16:0, C17:0, and C18:0.

^3^Unsaturated fatty acids: C14:1, C16:1, C18:1 *trans*-11, C18:1 *cis-*9, C18:2 n-6, C18:3 n-3, and CLA *cis*-9, *trans*-11.

^4^Monounsaturated fatty acids: C14:1, C16:1, C18:1t11, and C18:1 *cis-*9.

^5^Polyunsaturated fatty acids: C18:2 n-6, C18:3 n-3, and CLA *cis*-9, *trans*-11.

^6^C6:0, C8:0, C10:0, C12:0, and C14:0.

^7^C14:1 *cis*-9/(C14:0 + C14:1 *cis*-9).

^8^C16:1 *cis-*9/(C16:0 + C16:1 *cis*-9).

^9^C18:1 *cis-*9/(C18:0 + C18:1 *cis-*9).

^10^ CLA *cis*-9, *trans*-11/(C18:1 *trans*-11+ CLA *cis*-9, *trans*-11).

The estimates of the h_g_^  2^ are based on a univariate model.

### Heritability

SNP-based estimates of heritability and posterior standard deviations are presented in Table 1. Most of the heritability estimates were found to be moderate, with a range from 0.07 to 0.34. C17 was estimated to have the lowest heritability (0.07) among SFA. In contrast, the highest heritability among SFA was estimated for C8:0 and C10:0 (0.34). For UFA, the variation in heritability was smaller compared to the SFA. For UFA heritability, estimates ranged from 0.09 (C18:1 t11) to 0.26 (C14:1). Summarizing the individual FAs into the two groups of SFA and UFA resulted in heritability estimates of 0.09 for all SFA and 0.33 for all UFA. The heritability for the even chain SFA from C6:0 to C14:0 was 0.35. Splitting UFA into two groups of monounsaturated FAs (MUFA) and polyunsaturated FAs (PUFA) and comparing them to estimates of all UFA the heritability estimates of MUFA was 0.34; while, the heritability estimates for PUFA was 0.28. The four FA indices had heritability estimates from 0.16 to 0.35. For the four desaturase indices, the highest heritability was estimated for the C14 (0.31) and C18 (0.35) indices. For the C16 and CLA index, heritability was estimated to be 0.19 and 0.16, respectively. For all estimates of the individual FAs, the posterior standard deviations were relatively similar, with a range from 0.06 to 0.19. Similarly, the posterior standard deviation ranged from 0.08 to 0.20 for groups of FAs and from 0.12 to 0.19 for FA indices.

### Environmental correlation

Environmental correlation for single FAs is presented in Table 2. For SFA, there was a moderate to high positive environmental correlation for C6:0 to C16:0. A low negative environmental correlation was found between C16:0 and C17:0 (−0.03). In addition, the C18:0 showed a negative environmental correlation to all other SFA, with the exception of C17:0. C18:1c9 showed negative correlation to C6:0 to C16:0 and positive correlation with C18:0 and C18:1 t1, but close to 0 correlation with C16:1c9 and C17:0. The range for the environmental correlation among SFA ranged from −0.32 (between C12:0 and C18:0) to 0.97 (between C10:0 and C12:0).

**Table 2:** **Environmental**^**1**^**(below diagonal) and genomic**^**2**^**correlation (above diagonal) for individual milk fatty acids**^**3**^

**Trait**	**C6:0**	**C8:0**	**C10:0**	**C12:0**	**C13:0**	**C14:0**	**C15:0**	**C16:0**	**C17:0**	**C18:0**	**C14:1c9**	**C16:1c9**	**C18:1 t11**	**C18:1c9**	**C18:2n6**	**C18:3n3**	**CLA:c9t11**
C6:0		0.86	0.70	0.56	0.14	0.42	0.14	−0.02	−0.10	−0.11	−0.01	0.04	−0.07	−0.24	−0.02	0.06	−0.11
C8:0	0.90		0.86	0.75	0.21	0.47	0.17	−0.02	−0.12	−0.18	−0.04	0.06	−0.16	−0.22	0.00	0.01	−0.21
C10:0	0.65	0.89		0.91	0.33	0.47	0.24	0.00	−0.20	−0.22	−0.12	0.16	−0.17	−0.28	−0.04	−0.06	−0.27
C12:0	0.52	0.80	0.97		0.28	0.48	0.25	0.01	−0.18	−0.26	−0.06	0.14	−0.14	−0.20	−0.02	−0.06	−0.23
C13:0	0.26	0.46	0.53	0.59		0.23	0.49	0.05	0.01	−0.21	−0.23	0.14	−0.18	−0.10	0.00	−0.13	−0.27
C14:0	0.49	0.65	0.81	0.84	0.41		0.35	0.08	−0.25	−0.37	0.05	0.09	−0.08	−0.21	−0.04	0.03	0.00
C15:0	0.17	0.34	0.39	0.45	0.83	0.39		0.17	−0.12	−0.27	−0.15	0.13	−0.17	−0.23	−0.19	−0.29	−0.19
C16:0	0.19	0.04	−0.02	0.00	0.08	0.06	0.07		−0.07	−0.18	0.10	0.12	−0.17	−0.26	−0.19	−0.05	−0.05
C17:0	0.06	0.04	0.00	−0.02	0.14	0.00	0.32	−0.03		0.26	−0.29	−0.18	0.09	0.16	0.13	0.09	−0.02
C18:0	−0.11	−0.16	−0.22	−0.32	−0.20	−0.36	−0.20	−0.38	0.25		−0.14	−0.37	0.09	0.14	0.13	0.15	−0.07
C14:1c9	−0.01	0.09	0.12	0.27	0.36	0.34	0.53	0.17	−0.02	−0.68		0.07	−0.05	0.06	0.08	0.08	0.19
C16:1c9	−0.20	−0.20	−0.25	−0.12	0.17	−0.13	0.24	0.46	0.00	−0.43	0.55		−0.03	−0.09	−0.12	−0.17	−0.03
C18:1 t11	−0.29	−0.34	−0.34	−0.34	−0.10	−0.30	−0.12	−0.23	0.02	0.27	−0.06	−0.01		0.11	0.10	0.09	0.23
C18:1c9	−0.59	−0.65	−0.67	−0.64	−0.36	−0.57	−0.23	−0.65	0.01	0.27	−0.01	0.00	0.27		0.26	0.14	0.24
C18:2:6	−0.11	−0.14	−0.13	−0.16	−0.19	−0.10	−0.11	−0.35	0.01	0.05	−0.03	−0.03	0.28	0.40		0.63	0.37
C18:3n3	−0.04	−0.02	0.03	0.01	−0.04	0.01	−0.02	−0.27	0.02	−0.03	−0.03	−0.03	0.08	0.23	0.62		0.33
CLA:c9t11	−0.24	−0.23	−0.24	−0.22	−0.14	−0.16	−0.09	−0.30	−0.11	−0.03	0.16	0.12	0.59	0.47	0.31	0.15	

^1^Psd between 0.01 and 0.05.

^2^Psd between 0.06 and 0.10.

^3^Correlations are based on bivariate analysis.

### Genomic correlation

For single SFA even chain FAs (*i.e*., C6:0 to C14:0), there was a positive genomic correlation among the FAs, ranging from 0.42 to 0.91 (Table 2). For the uneven and long chain SFA, the genomic correlation with the other SFA was low. The complete range of correlation coefficients for SFA ranged from −0.37 (between C14:0 and C18:0) to 0.91 (between C10:0 and C12:0). FAs with similar structures tended to have a higher positive correlation with each other. The genomic correlation between SFA and UFA was −0.53 (Table 3). The individual UFAs have in general a negative genomic correlation with the other FAs. This was mainly negative with most of the short and medium even-chain SFA.

**Table 3:** **Estimates of environmental**^**1**^**(below diagonal) and genomic**^**2**^**(above diagonal) correlation for groups of fatty acids**^**3**^

**Trait**	**SFA**	**UFA**	**MUFA**	**PUFA**	**C6toC14**	**C14index**	**C16index**	**C18index**	**CLAindex**
SFA		−0.53	−0.60	−0.03	0.38	−0.25	−0.10	−0.11	0.07
UFA	−0.96		0.60	0.41	−0.22	0.26	0.01	0.16	0.08
MUFA	−0.95	0.99		0.32	−0.26	0.26	0.04	0.16	0.07
PUFA	−0.53	0.63	0.54		−0.04	0.16	−0.05	0.24	0.24
C6toC14	0.63	−0.68	−0.70	−0.20		−0.27	0.13	0.07	0.12
C14index	−0.19	0.31	0.33	0.07	−0.06		−0.01	0.16	0.41
C16index	−0.29	0.48	0.48	0.20	−0.25	0.67		0.24	0.09
C18index	−0.64	0.60	0.58	0.38	−0.12	0.70	0.62		0.37
CLAindex	0.02	0.01	0.00	0.10	0.19	0.13	0.13	0.39	

^1^Psd between 0.01 and 0.05.

^2^Psd between 0.05 and 0.11.

^3^Correlations are based on bivariate analysis.

For most single UFA, the estimated genomic correlation was negative (Table 2). The correlation coefficients between single UFA were found to range from 0 (C13:0 and C18:2n6) to 0.63 (between C18:3n3 and C18:2n6). In addition, relatively high correlations were found between C18:1c9, C18:2n6, C18:3n3, and CLAc9t11.

There was a positive correlation within groups of FAs consisting of either SFA or UFA, and a negative correlation between groups of SFA and UFA. Correlation between groups of FA ranged from −0.60 to 0.60 (Table 3). Genomic correlation between the four indices was in the range of −0.01 to 0.37 (Table 3). The lowest correlation was found between the C14 and C16 indices, which were estimated to be −0.01. For the C14 index, the correlation with the C18 and CLA indices was 0.16 and 0.41, respectively. Between C16 and C18 and between C16 and CLA, the correlation was estimated to be 0.24 and 0.09, respectively, and between C18 and CLA it was found to be 0.37.

**Figure 1:**
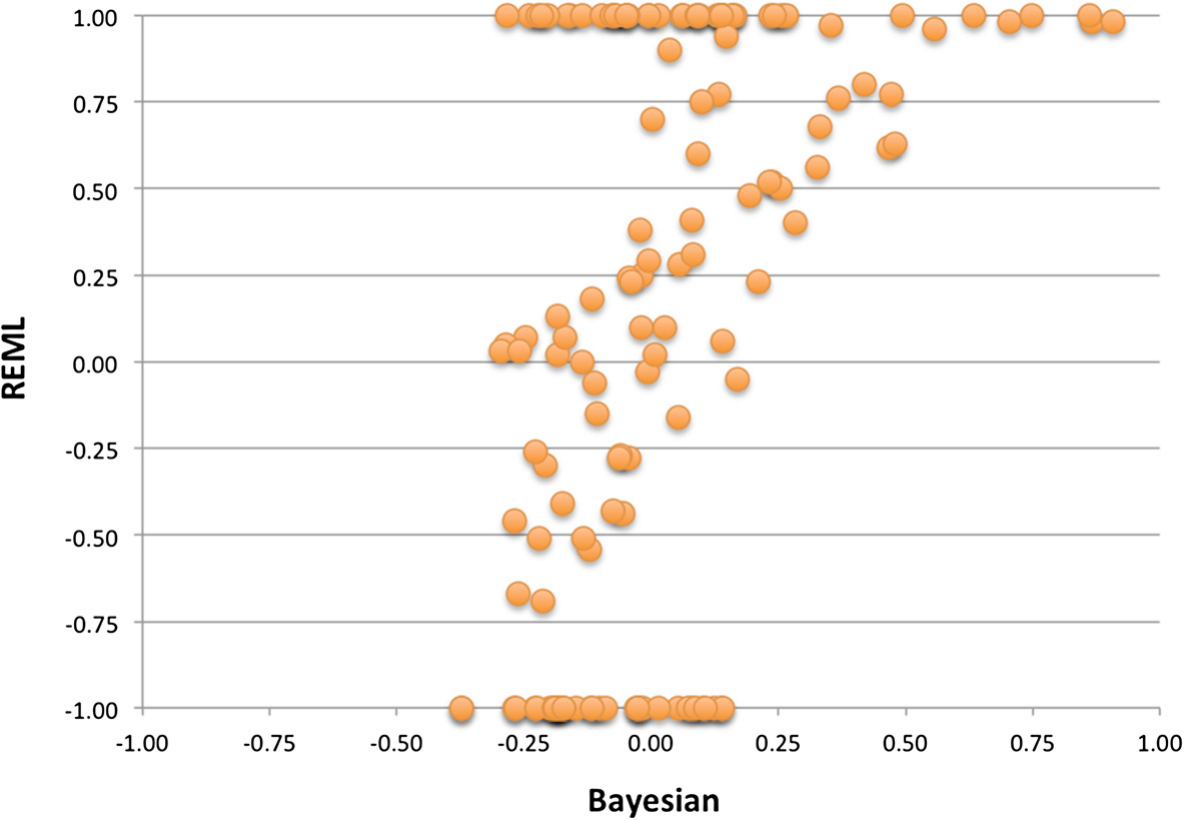
Genomic correlation estimates between the fatty acids based on a bivariate Bayesian model (x-axis) plotted against genomic correlation estimates between the fatty acids based on a bivariate REML model using a genomic relationship matrix (y-axis).

**Figure 2:**
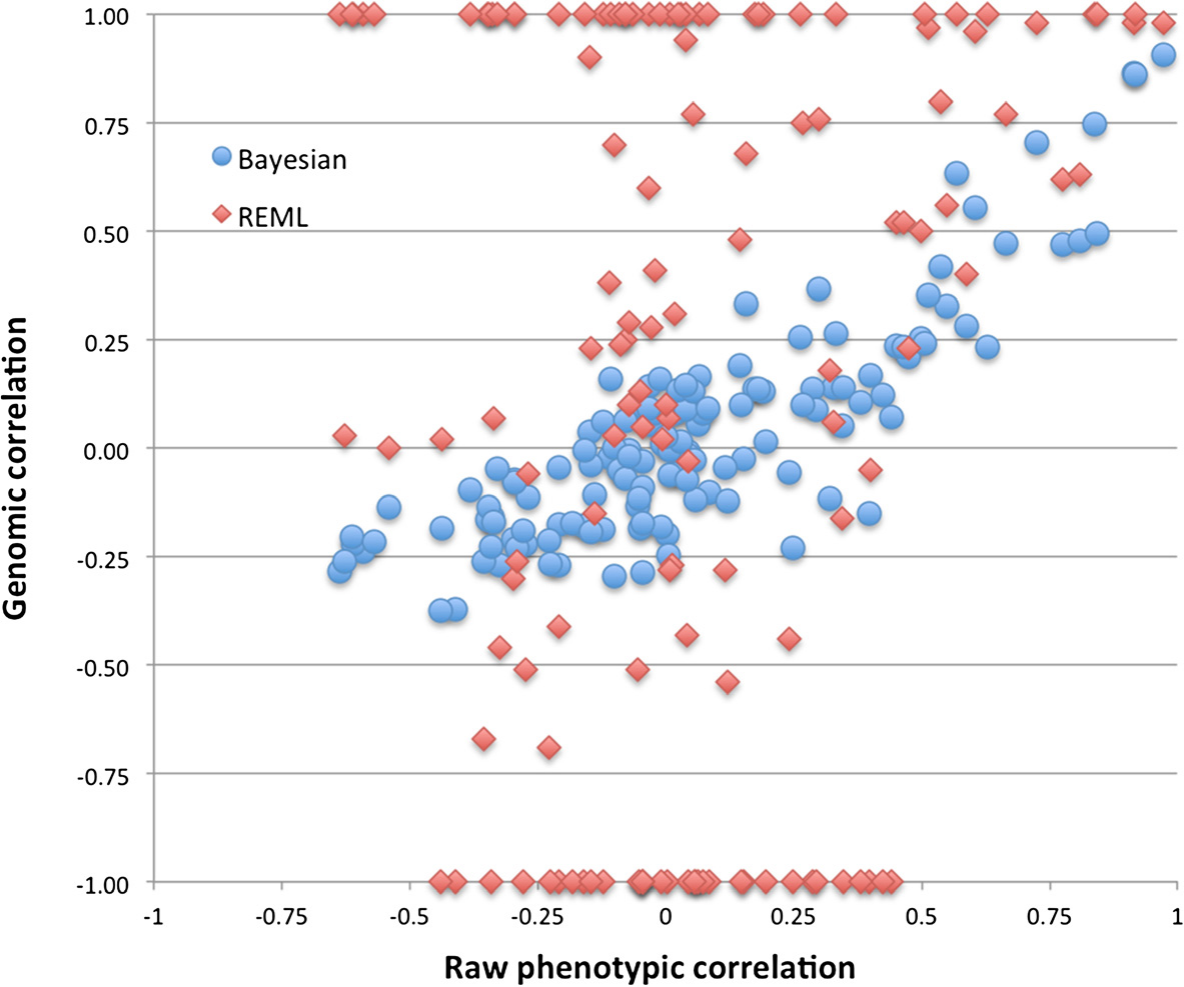
Raw phenotypic correlation between the fatty acids (x-axis) plotted against the genomic correlation between the fatty acids (y-axis) estimated using a bivariate Bayesian model (blue dots), and a bivariate REML model using a genomic relationship matrix (red diamante).

The results of the bivariate Bayesian models were verified using the same models in a REML analysis with a genomic relationship matrix as the random additive genomic effect (Appendix 1). Figure 1 shows the comparison of the genomic correlation between the Bayesian and REML approach. Figure 2 shows the correlation between the phenotypic correlation and the genomic correlation estimated the Bayesian and REML approach.

## Discussion

In this study, we present genetic parameters for specific FAs in Danish Holstein cattle using SNP markers and a Bayesian approach. Previous studies have presented heritability and correlation estimates for FAs from Holstein populations. However, all of these studies used a restricted maximum likelihood (REML) approach with pedigree information [3,10,12]. In contrast the population structure of this study consists of many sires with few offspring each which is well suited for association mapping but may compromise estimation of genetic parameters using pedigree. The use of the genomic relationship instead of a pedigree relationship could potentially give a better estimate of the relationship between the animals and therefore improve the possibility to estimate genomic parameters. Stoop et al. [4] estimated heritabilities for FAs from 1,918 records using pedigree and obtained standard errors on heritabilities in the range 0.07 to 0.12. The Bayesian posterior standard deviations on our SNP-based heritability estimates are comparable (0.06 to 0.19) but are based on only 371 records. To the best of our knowledge, the approach that we present in this study is novel.

### Heritability

Variability in the size of the heritability estimates was found when this study was compared to previous studies. Stoop et al. [4] presented heritability estimates from Dutch Holstein cattle that, in general, were higher than in our study. However, the pattern in the heritability estimates for single FAs was the same as in our study, where the FAs that had the highest and lowest heritability estimates in the study by Stoop et al. [4] were also found to have the highest and lowest heritability estimates in our study. In Stoop et al*.*[4], the highest heritability was estimated for C10:0 (0.54) whereas the lowest heritability was estimated for C18:3 *cis*-9 and C18:1 *trans*-11 (0.11 and 0.09, respectively). In this study, the highest heritability was estimated for C8:0 and C10:0 (both 0.34) and the lowest was estimated for C17:0 (0.07). In agreement with the results of Stoop et al. [3] the general pattern in our study was that individual SFA had the higher heritability estimates compared to individual UFA. This was also observed in the evaluation of groups of FAs. The study by Stoop et al*.*[4] estimated heritability to be 0.49 for a group consisting of even-chain FAs (from C6:0 to C12:0), whereas estimates for unsaturated C18 FAs was found to be 0.18. In our study, we estimated heritability to be 0.28 for a group consisting of even-chain SFA (from C6:0 to C14:0), and heritability for all UFA grouped together was 0.33. Some of the estimated heritabilities for individual FA by Garnsworthy et al. [12] were similar to the heritabilities presented in this study, e.g. C6:0, C8:0, C14:1, C18:1, whereas the hertitabilities for C12:0, C14:0, C16:0, C18:1 *cis*-9 and C18:1 *trans*-11 were lower in [12] compared to our study. The heritabilities for the SFA presented in [11] was higher compared to our study, while the heritability estimates for PUFA and MUFA and the group C6:0 to C14:0 presented in [12] were lower compared to our study.

In studies by Bobe et al*.*[10] and Mele et al*.*[3], estimates of heritability for FAs were determined for an American and Italian Holstein population, respectively. The pattern and size of heritability from these studies were different from our study and the study by Stoop et al. [4]. Bobe et al. [5] showed heritability estimates ranging from 0 to 0.49. These estimates were based on 233 animals coming from one experimental farm. The estimates are therefore not very accurate, which makes direct comparison to heritability estimates of other studies difficult. However trends in the heritability estimates can be used to compare to the results of other studies. The highest heritability estimates for SFA was for C8:0 and C10:0 (0.18 and 0.22, respectively), whereas the highest heritability for UFA and all FAs was estimated for C16:1 (0.49). Mele et al. [3] showed only a few heritability estimates for SFA, but the pattern in that study was in agreement with that of Bobe et al. [10], where heritability estimates for UFA were higher than for SFA. The FA indices, estimated by Mele et al*.*[3] were similar to our estimates, with the exception of the C16 index (0.12 vs. 0.31, respectively). This could be explained by the proportion of C16 in the milk within the two different populations. In contrast to other short and medium chain SFA, C16 is not only synthesized within the mammary gland, but also partially obtained from food. During the lactation period, the *de novo* synthesis and expression of C16 is altered. As C16 is by far the most common FA in bovine milk, alterations in expression can also alter the proportion used for the estimation [3,8].

### Missing heritability

In Krag et al. [11] we showed that the genomic heritability could be captured using a Bayesian or REML SNP based estimation of genomic heritabilities when we assumed evenly distributed QTL over the genome with similar effect. This is not the case in reality therefore we expect genomic heritabilities may be somewhat lower than pedigree-based heritabilities. This was also shown in Yang et al. [13]. They argued that insufficient LD with causal variants causes incomplete recovery of genomic variance and therefore underestimating the true heritability. Because in cattle the LD is higher than in human and we are using a high density SNP array, we expect the underestimation of the genomic heritability is less extreme as in e.g. human data.

### Phenotypic and genotypic correlation

In this study, we found a negative phenotypic correlation between groups of SFA and UFA and a positive correlation within the groups of SFA and UFA. The phenotypic correlation coefficient ranged from −0.95 (between SFA and UFA) to 0.99 (between MUFA and UFA). These estimates are similar to those previously presented by Bobe et al*.*[10]. The estimates for genetic correlation of our study were found to be substantially smaller than the estimates of phenotypic correlation, but the pattern was the same. The genetic correlation between UFA and SFA ranged from −0.15 to 0.15. In general, this range was larger, and the correlations were higher/lower, respectively, compared to the genetic correlation between single UFA.

For short and medium chain FAs (C6:0 to C14:0, with the exception of C13:0), the correlation was positive. The phenotypic correlation was high, whereas the genetic correlation was moderate. Stoop et al*.*[4] also reported a positive genetic correlation coefficient among single SFA; however, the values were somewhat higher. C18:0 had a phenotypic and genetic correlation with the other SFA. Most SFA are synthesized *de novo* within the mammary glands, and the synthesis is strongly regulated by activities of acetyl coenzyme A (CoA) carboxylase [8]. However, this is not the case for C18:0, which is derived from feed, and C16:0, which is synthesized in the mammary gland and derived from feed [14]. This explains the negative correlation between C18:0 and SFA and the positive correlation between C18:0 and UFA. Moreover, C16:1, C18:1, C18:2, and trans-fatty acids are all known to suppress the *de novo* synthesis of SFA [8].

### Possibilities for selection

The estimates that we have presented in this study indicate that it may be possible to alter the composition of FAs in bovine milk. The phenotypic correlation and some of the genetic correlations indicate that the FAs from this study can be subdivided into two groups, consisting of feed-derived FAs (mainly UFA) and those under higher genetic influence (short and medium chain SFA). Due to the negative correlation between groups of SFA and UFA, it may be possible to reduce the concentration of the less healthy SFA and increase the concentration of UFA.

The most common FAs identified in this study were C14:0, C16:0, C18:0, and C18:1 *cis*-9, which is also true for bovine milk in general. They comprise approximately 70% of the total FAs. Based on our results, we postulate that there is merit in increasing the proportion of C18:1 *cis-*9. An increase in the FA would be expected to reduce the amount of C14:0 and C16:0 expressed as well. C18:1 *cis-*9 showed a negative genetic correlation with both C14:0 and C16:0, but no genetic correlation with C18:0. We also observed a negative correlation between C18:0 and C18:1 *cis-*9, but this is not as large as the negative phenotypic correlation between C18:1 *cis-*9 C14:0, or C16:0. The general expectation would be that aiming to increase C18:1 *cis-*9 would result in a reduction in all other FAs, including both SFA and UFA. C16:0 is the most abundant FA in milk and has a low genetic correlation with the other FAs. This would be advantageous for selection, as this makes it possible to work specifically on a reduction of the concentration of C16:0 without affecting other FAs.

## Conclusions

In this study, we have presented heritability and correlation estimates for different FAs in Danish Holstein milk based on an analysis of SNP markers using a Bayesian approach. The heritability estimates obtained in this study are in general accordance with heritability estimates from studies using pedigree data and/or a genomic relationship matrix in the context of a REML approach. This indicates that it is possible to estimate heritability based on a random sampling from the population using SNP markers only based on a Bayesian approach. SFA and UFA expressed a strong negative phenotypic correlation and a weaker genetic correlation. This is in accordance with the theory that SFA is synthesized *de novo*, while UFA can be regulated independently from the regulation of SFA by the feeding regime.

## Methods

### Animals

The overall experimental strategy underlying this study was to minimize the potential sources of environmental variation and maximize the genetic variation in the sample population. For this purpose morning milk samples were collected from 371 Danish Holstein cows from October to December 2009 (winter period) from 19 herds. Between 3 and 24 milk samples were collected from individual cows of each herd. Selection of animals was designed to include as many unrelated animals as possible (*i.e*., maximizing the number of sires). In total, the 371 cows were sired by 200 bulls. All cows were housed in loose housing systems, fed according to standard practice, and milked twice a day. The cows sampled were all in mid-lactation (day 129 - day 227 of lactation) and within the first-third parity. Immediately after milking, the milk samples were placed on ice for transport to the laboratory.

### Fat and fatty acids

Fat content was determined in the fresh full milk samples using MilkoScan FT2 (FOSS, Hillerød, Denmark) and expressed as g/100 g milk. Cream was separated from skim milk by centrifugation (2643 × g for 30 min at 4°C). The cream samples were then stored at −20°C until analysis of FA composition using gas chromatography, essentially as previously described [15].

Peak areas for individual FA were calculated after gas chromatography separation, and FAs were identified and quantified through the use of external standards (Supelco 37 component FAME mix, Supelco, Bellafonte PA and GLC 469 methyl ester standard, Nu-Chek Prep Inc, Elysian, MN) and expressed as the weight proportion of total FA. Only FAs present at a minimum of 1 g/kg of FA (average) were included. Desaturase indices were calculated as the ratio between the product and the sum of the product and substrate, and were used as a proxy for delta-9-desaturase activity for the following pairs of product and substrate: C14:1 *cis*-9 and C14:0; C16:1 *cis*-9 and C16:0; C18:1 *cis*-9 and C18:0; and C18:2 *cis*-9, *trans*-11 and 18:1 *trans*-11. For a detailed description, please see [16].

### SNP Markers and Genotyping

In total, 371 animals was genotyped using the bovineHD beadchip [17]. Genomic DNA was extracted from ear tissue. From these animals, 777,962 SNP markers were assayed, with a median interval of 2.68 kb between SNPs (http://www.illumina.com/documents/products/datasheets/datasheet_bovineHD.pdf). The platform used was an Illumina® Infinium II Multisample assay device. SNP chips were scanned using iScan and analyzed with the software Beadstudio v. 3.1 (Illumina). The quality parameters used for selection of SNPs had minimum call rates of 80% for individuals and 95% for loci. Marker loci with minor allele frequencies (MAF) below 1% were excluded. The minimal acceptable GenCall score [18] was 0.65 for individual typing, and individuals with average GenCall scores below 0.65 were excluded. A total of 588,528 SNPs spread over BTA1-BTA29 were used in the analysis. The SNP positions within a chromosome were based on the *Bos taurus* genome assembly (Btau_4.0) [19].

### Bayesian mixed model

In this analysis, estimation of genetic parameters was based on phenotype and SNP data. The variance components were estimated with a Bayesian mixed model with a random regression version of a SNP-BLUP model using the software Bayz [20]. In matrix notation, the general mixed model can be described as:

$$y=\mu +X_{1}\beta_{1}+X_{2}\beta_{2}+\mathrm{Zu}+e$$

where ***y*** are the phenotypes, ***μ*** is a mean, and ***β***_***1,***_ and ***β***_***2***_ are vectors containing the effects for herd and parity. ***u*** is a vector with SNP effects taken as regression coefficients for allele substitution for each SNP in the study. ***X***_***1***_ and ***X***_***2***_ are incidence matrices for the fixed effects (herd and parity). Herd contained 20 levels and parity contained 3 levels. ***Z*** is a covariate matrix for the random effects (SNP markers) containing centred SNP covariates. Residuals are denoted ***e*** and are normal distributed with $e\sim N\left(0,I\sigma_{e}^{2}\right)$. In addition, the vector ***u*** containing the SNP effects is assumed to have a normal distribution with $\;u\sim N\left(0,I\sigma_{u}^{2}\right)$. For the distribution of ***e*** and ***u***, the variance is taken to be unknown, but is applied with a uniform distribution. For estimation of environmental and genomic correlations, bivariate models were used with a correlation between residual vectors and SNP-effects across the traits. The approach for estimating parameters in Bayz is based on a Markov chain Monte Carlo (MCMC). Due to the high number of SNP markers, a Metropolis-Hastings sampler was implemented to accelerate the process. For the MCMC, a total of 100,000 iterations were applied. Of these, the first 10,000 iterations was set as burn-in and therefore discarded. Only every 100^th^ estimate was saved for later analysis.

### Heritability and correlation estimation

A univariate Bayesian mixed model was used to estimate the heritability of the individual fatty acids, index traits and groups of fatty acids. A bivariate Bayesian mixed model was used to estimate the genomic and environmental correlations between the traits. Estimates of heritability and genetic correlation were retrieved from post-analyses made with the tool gbayz, which is part of the Bayz software [20]. Heritability was calculated as

$$h^{2}=\frac{\sigma_{a}^{2}}{\sigma_{a}^{2}+\sigma_{e}^{2}}$$

where $\sigma_{a}^{2}$ is the additive genomic variance computed as ***Var***(***Zu***) and $\sigma_{e}^{2}$ is the residual variance. The genomic and environmental correlation was calculated as:

$$r=\frac{\mathrm{Cov}\left(Zu_{1},Zu_{2}\right)}{\sqrt{\mathrm{Var}\left(Zu_{1}\right)*\mathrm{Var}\left(Zu_{2}\right)}}$$

where ***Cov***(***Zu***_1_**,*Zu***_2_) is the genomic covariance between trait 1 (***Zu***_1_) and trait 2 (***Zu***_2_), and ***Var***(***Zu***_1_) is the variance for trait 1 and ***Var***(***Zu***_2_) is the variance for trait 2. Genomic variance and genomic covariances are based on evaluating SNP-explained variance and covariance in each Gibbs cycle and averaged after all cycles had finished.

The results of the bivariate Bayesian models were verified using the same models in a REML analysis with a genomic relationship matrix as the random additive genomic effect (Appendix 1).

## Appendix 1

### Verification of the Bayesian approach using REML with a genomic relationship matrix

The calculation of the G-matrix, the estimation of the genomic heritability and the estimation of genomic correlations using a bivariate model were previously described in Buitenhuis et al. [21].

### Calculation of the G-matrix

The genomic relationship matrix was calculated for each chromosome separately as described by the first method presented in [22]. In short, let **M** be a matrix with dimensions of the number of individuals (*n*) by the number of loci (*m*) that specifies which marker alleles each individual inherited. The elements of **M** were set to −1, 0, 1 for the homozygote, heterozygote and the other homozygote, respectively. The diagonals of **M’M** counts the number of homozygous loci for each individual and off diagonals measure the number of alleles shared by relatives. Let the frequency of the second allele at locus *i* be *p*_*i*_, and let **P** contain the allele frequencies, such that column *i* of **P** equals 2(*p*_*i*_-0.5). Subtraction of **P** from **M** gives **Z**, which is needed to set the expected mean value to 0. The genomic relationship matrix **G** was then calculated as **ZZ´**/[2∑p_i_(1-p_i_)] [2].

### Estimation of genomic heritability and genomic correlations

To estimate the genetic parameters and variance components the REML approach in DMU was used [23]. The following model was used in the analysis:

(1) $$Y_{\mathrm{ijk}}=\mu +{\mathrm{herd}}_{i}+{\mathrm{parity}}_{j}+{\mathrm{animal}}_{k}+e_{\mathrm{ijk}}$$

Where Y_ijk_ is the phenotype of individual k in herd i and lactation j, μ is the fixed mean effect, herd is a fixed effect (i = 1, 2, …, 20), parity is a fixed effect (j = 1,2,3), and animal are the random genomic values with distribution N(0,**G**σ^2^_  a_). The residuals are assumed distributed N(0,**I**σ^2^_  e_).

Univariate analyses were performed to estimate the genomic heritability, which was defined as:

(2) $$h^{2}=\sigma^{2}{}_{a}/\left(\sigma^{2}{}_{a}+\sigma^{2}{}_{e}\right)$$

where σ^2^_   a_ was the genomic variation and σ^2^_   e_ was the residual variation. The genetic and phenotypic correlations were studied by fitting a series of bivariate analyses with a REML approach in DMU [23].

### Results

The genomic correlations estimated by the Bayesian approach are compared to the estimates from the REML approach in Figure 1. The estimates using the REML approach follows the estimates from the Bayesian approach even though a large part of the REML estimates are +1 or −1. In the cases the REML estimate goes to 1 or −1 there is a convergence problem.

Figure 2 shows the relation between the phenotypic correlation and the genomic correlation estimated by the REML approach and the Bayesian approach. While the large part of the REML estimates shows correlations between 1 and −1 and therefore a clear trend between the phenotypic and genomic correlation is missing, the Bayesian estimates show a positive trend between the phenotypic and genomic correlation. Assuming variances and covariances are (approximately) equally split in genomic and residual within and between traits, the phenotypic, genomic and residual correlations are also (approximately) equal. Therefore, the phenotypic correlation is a reasonable first proxy for the genomic and residual correlation, and over many bivariate analyses it is reasonable to assume that genomic correlations should roughly follow the phenotypic correlations. This is the pattern we see from the Bayesian genomic correlation estimates.

### Conclusion

Where the REML approach gives many correlations of either 1 or −1, the Bayesian approach follows the trend of REML estimates where the correlations is between 1 and −1. In cases where the REML analysis shows convergence problems (i.e. when correlations go to 1 or −1) the Bayesian approach is still giving a correlation.

## Competing interests

The authors declare no competing interests.

## Authors’ contributions

KK performed the genetic analysis and wrote the manuscript. NAP and MKL performed the extraction and analysis of the fatty acids from the milk samples and contributed to the discussion of the results. LBL contributed to the planning and discussion of the results. LLJ contributed to the genetic analysis of the data and contributed to the discussion. BB processed the genotypes and contributed to the genetic analysis of the data, discussion of the results and planning. All authors contributed to the manuscript and approved the final version of the manuscript.
